# Caesarean Section Rates and Activity-Based Funding in Northern Norway: A Model-Based Study Using the World Health Organization's Recommendation

**DOI:** 10.1155/2018/6764258

**Published:** 2018-07-16

**Authors:** Jan Norum, Tove Elisabeth Svee

**Affiliations:** ^1^Department of Surgery, Hammerfest Hospital, Hammerfest, Norway; ^2^Department of Clinical Medicine, Faculty of Health Science, UiT-The Arctic University of Norway, Tromsø, Norway; ^3^Department of Obstetrics, University Hospital of North Norway, Harstad, Norway

## Abstract

**Objective:**

Caesarean section (CS) rates vary significantly worldwide. The World Health Organization (WHO) has recommended a maximum CS rate of 15%. Norwegian hospitals are paid per CS (activity-based funding), employing the diagnosis-related group (DRG) system. We aimed to document how financial incentives can be affected by reduced CS rates, according to the WHO's recommendation.

**Methods:**

We employed a model-based analysis and included the 2016 data from the Norwegian Patient Registry (NPR) and the Medical Birth Registry of Norway (MBRN). The vaginal birth rate and CS rates of each hospital trust in Northern Norway were analyzed.

**Results:**

There were 4,860 deliveries and a 17.5% CS rate (range 13.9–20.3%). The total funding of the deliveries was €16,351,335 (CS: €6,389,323; vaginal births: €9,962,012). The CS rate varied significantly and was lower in the southern region (*P* < 0.002). Consequently, the introduction of a cutoff at a 15% CS rate would gain the two southern hospital trusts by a budget increase of 0.2%. The two northern ones would experience 6.4% less resources. A total of €644,655 could be allocated to further quality and safety initiatives in obstetrics.

**Conclusion:**

The economic consequences of the model-based financial incentive were low, but probably sufficient to get the necessary attention and influence on the CS rate.

**Recommendations:**

A financial incentive for the reduction of CS rates should be tested as a supplement to other instruments.

## 1. Introduction

Caesarean section (CS) rates have been significantly debated during the last years due to rising figures, significant variations, and the general focus on quality of care and patient safety. Despite the fact that CS rates above 15 percent seem to do more harm than good, rates have been reported up to more than 50 percent [1–4]. However, in the Nordic countries, the figures have been lower, but rising. They have increased from 14.4%–16.4% in 2000 to 16.5%–20.7% in 2011, with the highest figures in Denmark [5].

In the struggle for improved quality and safety in health care, the CS rate has been selected one of the quality of care measures, so also in Norway [6, 7]. CS rate is an easily obtained measure for quality and safety, but it is also a superficial measure. The registration and follow-up of CS rates have revealed significant variations [8]. The right level of CS rate has not been documented. It is now thirty years since the World Health Organization (WHO) first recommended a CS rate of 10–15% and researchers have argued it is time to move on [9, 10]. The WHO's update in 2014 confirmed their prior recommendation and concluded CS rates higher than 10% were not associated with reductions in maternal and newborn mortality rates and argued that CS should only be undertaken when medically necessary [10].

A frequently employed initiative to reduce the rates has improved medical information to mothers and relatives about the risk of undergoing CS [1]. Other factors are women's wish and obstetrician's gender and volume [11]. Obstetricians performing fewer deliveries per year (than the median number) have been shown having a twofold increase in CS rate [11]. One study showed male obstetricians considering CS on the maternal request less problematic than their female colleagues, and the female obstetricians were more often in favour of copayment for such a request [12]. To reduce the CS rates, it has been recommended to focus on nulliparous women (particularly by reducing the number of elective CS in these women and by encouraging vaginal birth after caesarean delivery (VBAC)) [13, 14].

Despite several initiatives introduced, very few have looked at economic ones. The financing of Norwegian hospitals is based on patient-related activity figures and categorized into groups employing the diagnosis-related group (DRG) system [15]. Consequently, hospital trusts having a high CS rate receive more economic resources than the others do. We aimed to explore the variations in CS rates and elucidate the economic consequences of paying for the WHO's suggested rate in our region.

## 2. Materials and Methods

**Figure 1:**
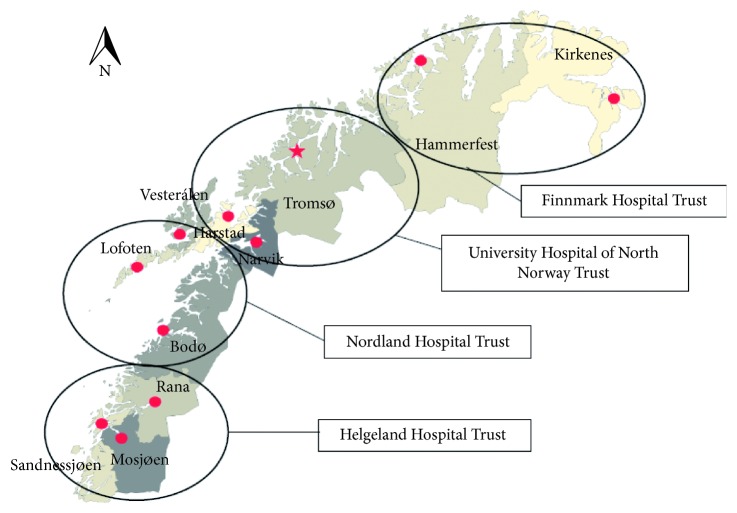
Four hospital trusts in Northern Norway.

In April 2017, we analyzed data from the Norwegian Patient Registry (NPR) on all deliveries in Northern Norway in 2016. In this region, there are four hospital trusts (Helgeland Hospital Trust, Nordland Hospital Trust, University Hospital of North Norway Trust, and Finnmark Hospital Trust). The two clinics of obstetrics and gynaecology are located at the Nordland Hospital in Bodø and at the University Hospital of North Norway in Tromsø. As the two clinics are comparable in their offer of obstetric service, the CS rate within the combined southern trusts (Helgeland and Nordland Hospital Trusts) can be compared with the northern (Finnmark and University Hospital of North Norway Trusts) ones. Their location is given in Figure 1.

According to regulations, all hospital trusts have to report their clinical activity to the NPR to get their funding by the Northern Norway Regional Health Authority (NNRHA) trust. The clinical activity is categorized according to the DRG system [15]. Consequently, the NPR database, among other data, includes each hospital trust's delivery data categorized according to the various DRGs. The connection between the reports of patient-related activity and financing (activity-based funding) ensures a complete registration, and this combining of activity and financing made the NPR figures the data of choice for our study. We accessed aggregated data available online at the NPR's website (https://helsedirektoratet.no/norsk-pasientregister-npr). The NPR was informed about the extraction of data, and they did give us advice during the process.

**Figure 2:**
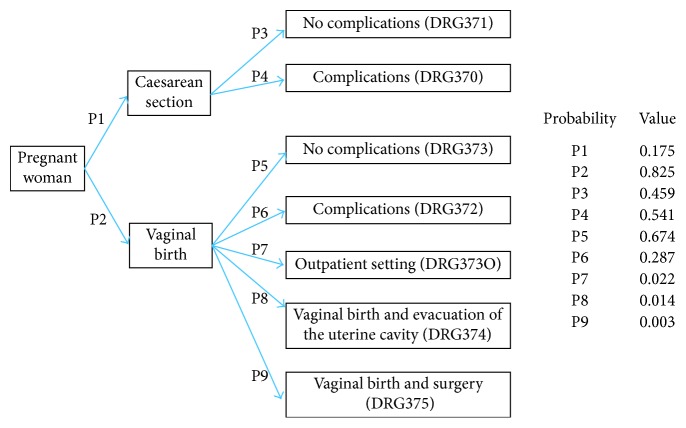
Probabilities (P1–P9) of undergoing different obstetric treatments during delivery in Northern Norway. The probabilities were based on the 2016 diagnosis-related group (DRG) data.

In delivery care, the actual DRGs are from DRG 370 to DRG 375. We added the values of each DRG according to the figures of 2016 [15]. Furthermore, the value was converted into euros (€) at a rate of 1 euro (€) = 9.3325 Norwegian krone (NOK), as of 16 May 2017 (http://www.norges-bank.no). An overview of the DRGs and the model is shown in Figure 2. The probabilities were based on the actual share of patients entering each DRG category during 2016 (NPR data). For example, a CS rate of 17.5% is a probability (P1) of CS of 0.175. Consequently, the probability (P2) of no CS was 0.825. Further details are given in Figure 2.

To clarify the robustness of the NPR data on delivery, we accessed the data registered in the Medical Birth Registry of Norway (MBRN) for the same period. All Norwegian hospital trusts have (according to law regulations) to report all deliveries to the MBRN. This information is available from the MBRN's web page (http://www.fhi.no/mfr) without any cost. The total number of deliveries and the number of CSs at each hospital trust were noted. We informed the MBRN about our study on CS.

### 2.1. Statistics and Ethical Permission

Individual data for each patient were recorded and analyzed by the NPR, and they did perform the quality assurance of the primary data. Microsoft Excel 2007 version was used for the final database and calculations. Anonymous and aggregated data from the NPR and MBRN were available on the web free of cost (open source). Groups were compared employing the Chi-square test. Significance was set to 5%.

The study was run as a quality of care analysis. Consequently, no consent of participation, consent for publication or ethical committee or data inspectorate approval was necessary. Similarly, no approval from the Regional Committees for Medical and Health Research Ethics (REK) or the Norwegian Social Science Data Services (NSD) was required.

## 3. Results

During study period, 4,860 deliveries were reported to the NPR, and a mean CS rate of 17.5% (range 13.9–20.3%) was noted. The number of deliveries in each hospital trust is given in Table 1. The 100% total DRG-based funding for the hospital trusts was €16,351,335 (€6,389,323 caesarean section and €9,962,012 vaginal births). Consequently, the mean funding per vaginal birth (4,010 births) was €2,484. Similarly, the funding per CS (850 CS) was €7,517. The corresponding delivery figures in 2016 taken from the MBRN were 4,003 vaginal births and 839 caesarean sections. Comparing NPR with MBRN (NPR/MBR), the figures for vaginal birth and CS were 4010/4003 = 1.0017 and 850/839 = 1.0131, respectively. These findings strongly indicate the NPR figures being robust.

A CS financing cutoff at a 15% rate caused various budget impacts. Although the Helgeland Hospital Trust would get €37,028 added to their budget, the other three trusts would experience a reduction of €681,683. The southern region (Helgeland and Nordland Hospital Trusts) would together gain resources (€11,323) while the northern region (University and Finnmark Hospital Trusts) would experience a reduction of €620,263. The released resources (€608,940) will not be lost, but kept at the “mother institution” of all hospital trusts (the NNRHA) for possible initiatives. An overview of the total figure and those of each hospital trust is shown in Table 2.

The CS rate of each hospital trust is given in Table 1. When exploring each hospital's CS rate, we noted the southern region (Nordland and Helgeland Hospital Trusts) having the lowest CS rate (14.6%) compared with the northern region (University and Finnmark Hospital Trusts) (18.5%) (*P*=0.0002).

## 4. Discussion

In this study, we have shown significant variations in CS rates between hospital trusts in a sparsely populated region (480,000 inhabitants) of Norway. The model-based economic effect of implementing a financial 15% cutoff in CS rate would increase the resources to the southern trusts and cause less funding to the northern ones. However, the total budget impact will be minimal.

The financial cutoff of at a CS rate of 15% was selected based on the recommendation of the WHO [9, 10, 16]. In 1985, participants at the WHO meeting held in Fortaleza, Brazil, stated that CS rates higher than 15% could hardly be justified from a medical standpoint [16]. Over the past three decades, health care professionals, scientists, epidemiologists, and policy makers have increasingly expressed the need to revisit the 1985 recommended rate [10]. In 2014, the WHO undertook a worldwide study to assess the association between caesarean section and maternal and neonatal mortality [17, 18]. The results were discussed by a panel of international experts at a consultation convened by the WHO in Geneva, Switzerland, in 2014. Based on the review, increases in caesarean section rates up to 10–15% at the population level were associated with decreases in maternal, neonatal, and infant mortality [18]. Above this level, increasing the rate of caesarean section was no longer associated with reduced mortality. However, the association between higher rates of caesarean section and lower mortality weakened or even disappeared.

We revealed a significant variation in CS rates within our region. This has also been observed worldwide. Data from 150 countries revealed that 18.6% of all births occur by CS, ranging from 6% to 27% [19]. However, several countries have reported higher figures within their own regions. In China, the rates of CS were 69.0%, 65.5%, and 59.2% in the three sample tertiary hospitals in Chongqing [3]. Brazil is also known for its very high CS rates, and figures of 51.9% have been published [1]. Among all countries reported by Molina et al. [2], Mexico and Chile had the highest CS rate, respectively, 46.9% and 49.6%. In Europe, Italy (36.8%) and Romania (36.3%) had the highest rate, whereas Cyprus and Finland had the lowest rate of 11.4% and 16.2%, respectively. In the Campania region of Italy, the figure reached 60.0% [20]. Caesarean delivery was the most commonly performed major surgery in the United States and accounted for approximately one-third of all deliveries [21]. Based on all these figures, the Northern Norwegian figure of 16.9% may look acceptable.

Whereas the figures of the Nordic countries are among the lowest rates in Europe, there are significant variations within the countries [5]. Pyykönen et al. [5] reported figures in 2011 ranging from 16.5% in Norway to 20.7% in Denmark. The increasing rate was explained by raised CS rates among nulliparous women and by an increased percentage of women with previous caesarean. Looking at Sweden, the CS rates varied significantly between counties and university hospitals [22]. Whereas the CS rate in 2014 was 21.6% in Stockholm County, the figure was 11.6% in Östergötland County. Furthermore, the Karolinska University Hospital in Solna had a CS rate of 23.0%, whereas the figure at the University Hospital in Linköping was only 7.5% [22]. Looking at Norway, especially the western region has traditionally reported a lower percentage (12.8%) [6]. For example, the figure of Haukeland University Hospital in Bergen was 11.7% in 2016, and they claimed that “safe delivery is bad business.” This is as they receive less funding due to a low CS rate. Similarly, we have documented that it is also “bad business” for our two southern hospital trusts (Helgeland and Nordland Hospital Trust) having the lowest rates.

Economic initiatives have shown to have influence on hospital treatment choices [23, 24]. There are strong reasons to believe the introduction of a CS financial cutoff level will reduce the health and financial burdens associated with this operation, both in the index and any future pregnancies. However, the economic incitements should be followed up by safe interventions. Nakamura-Pereira et al. recommended that public policies in Brazil should be directed at reducing CS in nulliparous women, particularly by reducing the number of elective CS in these women and encouraging vaginal birth after CS to reduce repeated CS in multiparous women [1]. Obstetrician volume of deliveries has documented another potentially modifiable risk factor for CS [11]. A twofold increase in the odds of caesarean delivery was revealed for patients whose obstetricians performed fewer than the median (60 deliveries/year) number of deliveries per year. Somewhat surprising, the obstetrician's years of experience did not have a similar effect. McClelland et al. at the Langone Medical Center in New York analyzed 37,692 deliveries and observed a mean CS rate of 29.6%, with a significant range for individual physicians from 9.9% to 75.6% [25]. In multivariate regression analysis, higher CS rate was directly correlated with patient age, physician male gender, proportion of high-risk deliveries, and maternal-fetal medicine specialty. Furthermore, it was inversely correlated with total number of deliveries by the physician and forceps delivery rate. Avoidance of unnecessary caesarean section has been a quality target [6]. To counteract the effect of the individual obstetrician, the requirement for a second obstetric opinion may be important [26]. Furthermore, patient and community education, clinical audit and feedback mechanism, clinical practice guidelines, and quality improvement strategies may be supportive alternatives to financial incentives. A Dutch study revealed pregnancy-related anxiety associated with primary caesarean section [23]. Women's wish for a CS may be due to anxiety, local culture/traditions, the “convenience for the doctor on duty,” and the aim of “fast deliveries on time.” It has been argued that women have been misled by the “grey literature” to believe it is less stressful to their babies, and less risky and more convenient to the mother herself to have a CS performed. In such a setting, the importance of respectful communication and maternity care (patient-centered approach) should not be forgotten [24]. Fuglenes et al. [12] documented that especially female obstetricians supported the use of copayment and higher copayments (up to €7.500) when CS should be performed on the maternal request and not on obstetric indications.

Because the individual obstetrician may influence on the CS rate, studies have focused on midwives' and doctors' own caesarean section rates [27, 28]. Whereas the Swedish study did not detect any significant difference between caesarean section as the mode of delivery for midwives and obstetriciansas compared to the general population, the Norwegian study revealed Norwegian female doctors and midwives having a higher CS rate than other professionals with an education of comparable duration [27, 28]. This may indicate that we have a homework to do in Norway. However, it should be noted that there was a ten years difference between the two studies.

In our study, we have documented a financial impact of employing a reduced and recommended CS rate. Yang et al. studied the relationship between malpractice litigation pressure and rates of CS and vaginal birth after CS (VBAC) [13]. They concluded that malpractice premiums are positively associated with rates of caesarean section and primary caesarean section and negatively associated with VBAC rates. They argue that a decrease in premiums for obstetrician-gynaecologists would be associated with an increase in the VBAC rate and decrease in the rates of caesarean section and primary caesarean section, respectively. The effect of economic incentives in this setting has also been documented in India [29]. A significant impact of the financial incentives on the choice of delivery method was revealed. Back in 1996, the Stockholm School of Economics indicated the additional cost of unnecessary CS in Sweden to be 12–14 million Swedish krone (SEK) (€1.3–1.5 million) [30].

The decision to perform a CS should be based on medical information and a valuation of the risk for the mother and foetus and not on economics [1]. However, economic incentives may be a supportive tool to reach the recommended level of CS rate. It may be argued that such an economic tool may be “unfair” to hospitals not getting a sufficient refunding according to their CS practice. However, various pricing has already been reported in the US [31]. Definitive evidence demonstrating a link between economic incentives and improved health outcomes is lacking. However, the evidence suggests that financial incentives can increase the quality of maternal health services. A Korean study showed continuous and marked improvement in the composite quality scores of the CS measures between 2007 and 2010 [32]. With the demonstrated success of the project, the Korean Ministry of Health and Welfare expanded the program.

When aiming for a reduction in caesarean delivery rate, the trends need to be monitored carefully [33].

## 5. Conclusions

The decision to perform a CS should be based on medical information and a valuation of the risk for the mother and foetus and not on economics. However, economic incentives may be a supportive tool to reach recommended level of CS rate. We recommend an economic incitement including a careful monitoring for possible unwanted “side effects.” Allocated resources should be used to increase the quality of maternal health services.

## Figures and Tables

**Table 1:** Deliveries in 2016 at each hospital trust in Northern Norway according to diagnosis-related groups (DRGs).

Hospital trust	Delivery	DRG	Helgeland Hospital		Nordland Hospital		University Hospital		Finnmark Hospital		Total	
			Number	%	Number	%	Number	%	Number	%	Number	%
Caesarean section	No complication	371	67	*10.6*	96	*6.8*	160	*7.6*	67	*9.4*	390	*8.0*
	Complication	370	21	*3.3*	119	*8.5*	268	*12.7*	52	*7.3*	460	*9.5*

Total caesarean section		—	88	*13.9*	215	*15.3*	428	*20.3*	119	*16.8*	850	*17.5*

Vaginal birth	No complication	373	405	*64.0*	768	*54.8*	1123	*53.1*	405	*57.0*	2701	*55.6*
	Complication	372	97	*15.3*	382	*27.2*	502	*23.7*	170	*23.9*	1151	*23.7*
	Outpatient	373O	18	*2.8*	19	*1.4*	43	*2.0*	8	*1.1*	88	*1.8*
	Evacuation	374	21	*3.3*	18	*1.3*	9	*0.4*	8	*1.1*	56	*1.2*
	Other surgery	375	4	*0.6*	0	*0.0*	10	*0.5*	0	*0.0*	14	*0.3*

Total vaginal birth			545	*86.1*	1187	*84.7*	1687	*79.7*	591	*83.2*	4010	*82.5*

Total			633	*100.0*	1402	*100.0*	2115	*100.0*	710	*100.0*	4860	*100.0*

Source: Norwegian Patient Registry (NPR).

**Table 2:** Effect of introducing a 15% limit of coverage for caesarean sections (CS) in hospital trusts in Northern Norway.

Hospital trust	Helgeland Hospital	Nordland Hospital	University Hospital	Finnmark Hospital	Total
DRG CS	661,483	1,616,123	3,217,212	894,505	6,389,323
DRG vaginal birth	1,353,939	2,948,855	4,191,001	1,468,217	9,962,012
15% CS	713,725	1,580,794	2,384,721	800,545	5,479,784
85% vaginal birth	1,336,673	2,960,531	4,466,137	1,499,270	10,262,612
Difference	−34,976	23,653	557,356	62,907	608,940

The figures are in euro (€).
